# Serum EGF-receptor and HER-2 extracellular domains and prognosis of non-small-cell lung cancer

**DOI:** 10.1038/sj.bjc.6601987

**Published:** 2004-06-29

**Authors:** W Jacot, J-L Pujol, J-M Boher, P-J Lamy

**Affiliations:** 1 Thoracic Oncology Unit, Centre Hospitalier Universitaire de Montpellier, Hôpital Arnaud de Villeneuve, 34295 Montpellier Cedex 5, France; 2 Department of Statistics and Epidemiology, University Institute for Clinical Research, Hôpital Universitaire Arnaud de Villeneuve, France; 3 Immuno-Radio-Assay Laboratory, Cancer institute, 34298 Montpellier Cedex 5, France

**Keywords:** non-small-cell lung cancer, extracellular domain, EGFR, HER-2, prognosis

## Abstract

This retrospective study aimed at evaluating the prognostic impact of high serum levels of either the HER-2 extracellular domain (ECD) or the epidermal growth factor receptor (EGFR) ECD measured using two specific ELISAs in 221 patients with non-small-cell lung cancer (NSCLC) receiving conventional therapy and 41 nonmalignant pulmonary diseases patients. It was not possible to discriminate between lung cancer and benign lung disease owing to the lack of sensitivity–specificity relationship of HER-2 and EGFR ECD levels. Neither HER-2 nor EGFR ECD specific levels were associated with a particular prognosis of NSCLC patients receiving conventional therapy.

Future development in non-small-cell lung cancer (NSCLC) therapy needs a better appraisal of prognostic determinants. Among the four tyrosine kinase receptors belonging to the HER family (Hirsch *et al*, 2003), the HER-2 oncogene encodes a protein with a molecular weight of 185 000D (p185) composed of three distinct portions: a cytoplasmic domain with tyrosine kinase activity, a transmembrane domain and an extracellular domain (ECD). The HER-2 ECD is a glycoprotein referred to as p105 that can be shed from the cell surface into the blood of normal individuals and can be elevated in different pathologic conditions (Molina *et al*, 1997). This ECD can be accurately quantified in serum with an enzyme-linked immunosorbent assay (ELISA) (Zabrecky *et al*, 1991). The HER-2 ECD releasing seems to be a process occurring in an early stage of pulmonary carcinogenesis (Brandt-Rauf *et al*, 1994). In lung adenocarcinoma, a study showed a relationship between serum HER-2 ECD level and HER-2 immunohistochemical detection in the tumour (Osaki *et al*, 1995). Another putative marker of NSCLC is the epidermal growth factor receptor (EGFR; HER-1), a 170 kDa membrane-bound tyrosine kinase receptor found on the surface of epithelial cells and implicated in cell growth regulation (Hirsch *et al*, 2003). Epidermal growth factor receptor is frequently overexpressed in NSCLC tumour cells, ranking from 65 to 84% (Bunn and Franklin, 2002). Serum EGFR ECD level could be a candidate tumour marker for diagnosis, EGFR-targeted therapies (Herbst, 2002) and prognosis of NSCLC. The EGFR ECD can be detected in the serum of healthy individuals and cancer patients, including NSCLC patients (Partanen *et al*, 1994). In this study, we examined the prognosis of a large NSCLC population followed up over a long period of time, simultaneously assessing serum HER-2 ECD and EGFR ECD together with classical prognostic determinants.

## PATIENTS AND METHODS

A total of 221 unselected and consecutive histologically proven and previously untreated NSCLC patients referred to the Montpellier – Nîmes University Hospital between January 1995 and December 1997 were entered into this study. Clinical data were prospectively recorded, and a blood sample was taken from each patient at presentation, the serum was separated and stored at −180°C (in triplicate) until tested. However, this study was retrospective in essence, as the decision to test HER-2 and EGFR ECD in the prospectively built sera bank was taken recently.

Histological subclassification was carried out according to the World Health Organisation (WHO) classification (WHO, 1982). Performance status was estimated according to the Eastern Cooperative Oncology Group (ECOG) criteria, and the percentage of weight loss during the previous 4 months was recorded. Staging was carried out according to the fourth edition of the UICC tumour node metastases (TNM) classification (Sobin *et al*, 1987), the American Thoracic Society map of regional pulmonary nodes (Tisi *et al*, 1982) and the new Mountain stage grouping (Mountain, 1997). The following investigations were carried out: clinical examination, standard chest roentgenography, computed tomographic (CT) scan of chest, brain and upper abdomen, fiberoptic bronchoscopy, liver sonography and bone scanning. Mediastinoscopy was used to establish nodal status in NSCLC patients with nonmetastatic disease and evidence of mediastinal lymph node enlargement on chest CT-scan.

Non-small-cell lung cancer patients with stage I or II, or resectable IIIa disease underwent surgery aimed at achieving complete resection. Other patients with performance status ⩽2 and unresectable stage IIIa or stage IIIb–IV disease received cisplatin-based chemotherapy. Radiotherapy was applied in locally advanced stage disease using a concomitant schedule. Best supportive care, including palliative radiotherapy when needed, was given to patients with advanced stage disease and poor performance status. Treatment was chosen according to clinical and routine biological findings and without knowledge of the serum EGFR and HER-2 levels. Hence, treatment was not considered as a prognostic variable in this study.

As a control group, the serum markers were measured in 41 consecutive patients with nonmalignant pulmonary diseases (infectious diseases 7%, chronic obstructive pulmonary diseases 12%, asthma 56% and miscellaneous 25%). The median age for the control population was 48 years and sex ratio (F/M) was 0.5.

Serum EGFR level was measured using the Oncogene Science EGFR Microtiter ELISA. This sandwich immunoassay uses two mouse monoclonal antibodies; the first one is coated to the solid phase, and the second, labelled using alkaline phosphatase, is used as a tracer. Both capture and detector reagents specifically recognise the EGFR ECD. The capture antibody recognises a protein domain on the extracellular portion of EGFR and neither inhibits EGF binding nor crossreacts with HER-2 oncoprotein. Standards are provided in the kit that allow accurate, quantitative determinations of EGFR in suitable samples. Serum HER-2 level was measured by the Oncogene Science HER-2 Microtiter ELISA. This test is a sandwich-type enzyme immunoassay that utilises two monoclonal antibodies directed to the HER-2 ECD. The assay quantitates either the full-length molecule in tumour tissue (p185) or the ECD (p105) in serum, plasma, cell cultures and fluids. Coefficients of variation of two sera tested in different assays were 4% for ELISA tests. Epidermal growth factor receptor and HER-2 Microtiter ELISA kits were kindly provided by AstraZeneca.

Receiver operating characteristic (ROC) curves were constructed using both patient and control subject serum marker levels in an attempt to establish a sensitivity–specificity relationship for both HER ECDs. Areas under the ROC curves (AUC–ROC) were calculated (Hanley and McNeil, 1982). Version 1.0 of AccuROC for Windows 95 software was used (Vida, 1993). The Youden indices (sensitivity + specificity −1) for each threshold value were generated using a Box–Cox transformation of the serum value of each marker in order to normalise the marker value (Box and Cox, 1964).

Survival data was updated in May 2003. At this time end point, nine patients were lost to follow-up (4%). Survival was defined as the time from treatment initiation (concomitant to serum sampling) to the date of death. Deaths related to the disease, regardless of the progression site, or related to treatment were analysed as events. Deaths from other causes were treated as censored observations. The median follow-up was 7 years and 4 months. Probability of survival was estimated by the Kaplan–Meier method (Kaplan and Meier, 1958). Single variable survival analyses were carried out by means of Wilcoxon and log-rank tests, and multivariate regression was carried out using Cox's model (Cox, 1972). Variables reaching at least a *P* level less than 15% in univariate analysis were tested in the Cox model. For each variable, the proportional hazards assumption was tested graphically. A *P* level of less than 0.05 was considered significant. The SAS software package was used.

## RESULTS

Patient characteristics are summarised in Table 1

**Table 1:** Patients characteristics and univariate analysis

**Variable**	**Number (%)**	**Median survival (weeks)**	***P* (log-rank)**
Age (year)			
⩽62		38.6	0.92
>62		39.7	
			
Gender			
Female	16 (7)	66.1	0.48
Male	205 (93)	35.3	
			
ECOG performance status			
<2	118 (53.5)	68.0	< 10^−4^
⩾2	102 (46.5)	23.7	
Not done	1 (0.5)		
			
Tumour status (T)			
1–2	62 (28)	47.4	0.0009
3–4	159 (72)	34.4	
			
Nodal status (N)			
0	58 (26)	97.6	< 10^−4^
1–3	163 (74)	31.4	
			
Metastatic status (M)			
0	116 (52)	50.4	<10^−4^
1	105 (48)	26.1	
			
Stage grouping			
I–II	22 (10)	351.7	< 10^−4^
III–IV	199 (90)	35.1	
			
Histology			
SQC	127 (57.5)	46.4	0.15
ADE	56 (25.5)	37.4	
LCC	38 (17)	23.7	
			
Weight loss			
<5%	146 (66)	43.7	0.18
⩾5%	66 (30)	26.9	
Not done	9 (5)		
			
Serum CYFRA 21-1 level			
⩽3.6	121 (55)	61.4	< 10^−4^
>3.6	100 (45)	26.6	
			
Serum NSE level			
⩽12.5	196 (88.6)	42.1	0.04
>12.5	25 (11.4)	20.9	
			
Blood leucocyte count			
⩽10.10^9^ l^−1^	113 (51.1)	57.3	0.0008
> 10.10^9^ l^−1^	105 (47.5)	28.4	
Missing data	3 (1.4)		
			
Serum albumin level			
⩾32 g/l	184 (83.2)	39.7	0.09
<32 g/l	32 (14.4)	17.7	
Missing data	5 (2.2)		
			
Serum sodium level			
⩾132	208 (94)	38.6	0.12
<132	12 (5.5)	27.3	
Missing data	1 (0.5)		
			
Serum alkaline phosphatase level			
Normal	191 (86.5)	42.1	0.004
Elevated	27 (12.2)	22.3	
Missing data	3 (1.3)		
			
Serum lactate dehydrogenase level			
Normal	110 (49.7)	39.0	0.53
Elevated	109 (49.3)	36.1	
Missing data	2 (1)		
			
Serum EGFR ECD			
⩽52 ng ml^−1^	96 (43)	28.4	0.23
>52 ng ml^−1^	125 (57)	44.7	
			
Serum HER-2 ECD			
⩽8.2 ng ml^−1^	115 (51)	37.3	0.30
> 8.2 ng ml^−1^	106 (49)	42.1	

EGFR=epidermal growth factor receptor; ECD=extracellular domain; NSE=neuron-specific enolase; ADE=adenocarcinomas; SQC=squamous cell carcinomas; LCC=large-cell carcinomas.

. Most of the main characteristics were similar to those expected for an NSCLC population, particularly the median age of 62 years (range 31–87; Interquartile Range: [52.3–70.2]).

### Non-small-cell lung cancer *vs* benign lung disease

Sensitivity was assessed in the NSCLC population and specificity in benign lung disease. Neither serum HER-2 nor EGFR ECD demonstrated any sensitivity–specificity relationship with both ROC curves underneath the noninformation line. AUC–ROC (standard deviation, 95% confidence interval) were 0.34 (0.04, 0.26–0.43) and 0.35 (0.05, 0.26–0.44) for HER-2 and EGFR ECD, respectively. According to the Youden indices, the best threshold value could be set at 8.2 ng ml^−1^ for serum HER-2 ECD, resulting in the maximum indices (0.23) and gave a 0.45 sensitivity and a 0.78 specificity. The best threshold value could be set at 52 ng ml^−1^ for serum EGFR ECD, resulting in the maximum indices (0.16) and gave a 0.44 sensitivity and a 0.72 specificity. Although these thresholds resulted in a poor specificity–sensitivity relationship, they were chosen as the cutoff level for subsequent survival analyses.

### Univariate analysis

**Figure 1:**
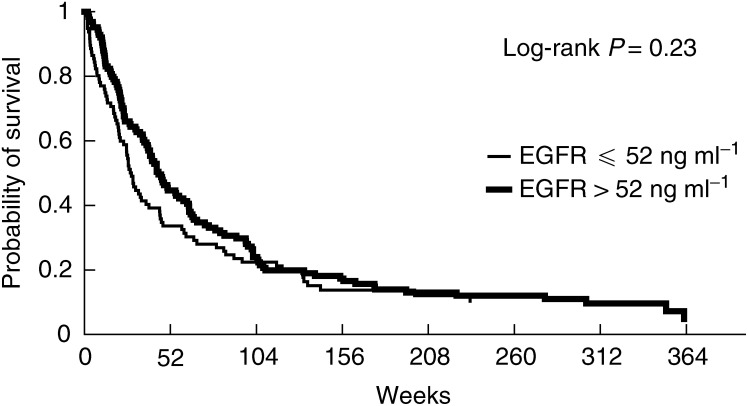
Probability of survival of NSCLC patients according to pretreatment serum HER-2 extracellular domain level.

**Figure 2:**
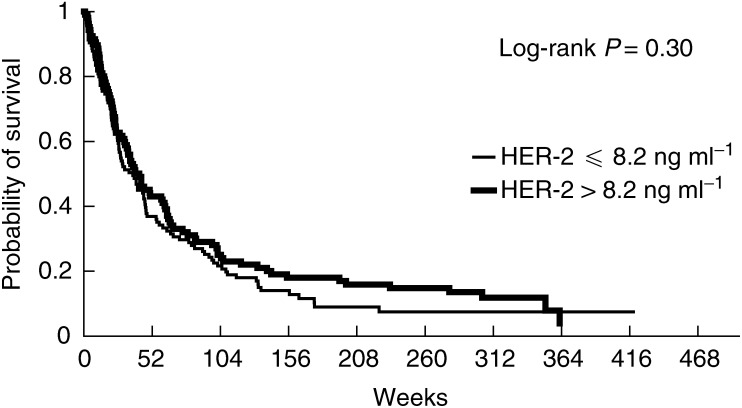
Probability of survival of NSCLC patients according to pretreatment serum EGFR extracellular domain level.

Univariate analysis (Table 1) showed that patients affected by one of the following characteristics had a shorter survival in comparison with the opposite status of each variable: performance status equal to or worse than 2, tumour status of T3–T4, positive nodal status (N1–N3), presence of metastases, stage grouping of III or IV, high serum alkaline phosphatase level, leucocyte count of 10 000 or higher, serum NSE level higher than 12.5 ng ml^−1^ and serum CYFRA 21-1 level higher than 3.6 ng ml^−1^. Neither serum HER-2 (Figure 1

) nor EGFR ECD (Figure 2

) were significant determinants of prognosis. However, the former was marginally significant when arbitrarily using the 15 ng ml^−1^ threshold as used in breast cancer populations (five out of 221 patients, log-rank test 0.07).

### Multivariate analysis

As the aim of the study was to determine the prognostic value of HER-2 and EGFR ECD serum levels, these variables were tested in the Cox model despite the lack of predictive value in univariate analysis. According to the Cox model, 12 additional variables were eligible to be tested in the Cox regression hazards model. They represented less than 10% of the total observed events (192 deaths) and therefore complied with the current recommendation (Harrell *et al*, 1985).

The following variables were independent determinants of a poor outcome: a poor performance status, hazard ratio (95 % confidence interval): 2.28 (1.59–3.27), a positive nodal status 2.0 (1.38–2.99), presence of metastases 1.48 (1.04–2.09), a nonsquamous histology 1.38 (1.01–1.90), a high serum CYFRA 21-1 level 1.5 (1.08–2.09), a high serum NSE level 2.28 (1.42–3.66) and an elevated alkaline phosphatase level 1.6 (1.01–2.54). Finally, the Cox model has been run again after coding HER-2 as a continuous variable. This variable did not modify the results of the Cox model.

## DISCUSSION

In a study of serum HER-2 ECD level in NSCLC (Ardizzoni *et al*, 2001), 20% (16 out of 82) of the patients presented with a high serum level and showed a shorter overall survival when compared with patients having a serum level < 73 fm ml^−1^ (13.5 ng ml^−1^). A major difficulty in analysing the HER-2 serum level literature relates to the inconsistency of threshold level definition. In our study, the lack of sensitivity–specificity relationship of serum HER-2 and EGFR ECD levels preclude a clear definition of the cutoff level to be subsequently used in a clinical setting. Therefore, it was not possible to discriminate between lung cancer and benign lung disease. The variables shown in this study, to be independent determinants of a poor outcome, are well-established survival features of NSCLC (i.e. poor performance status, positive nodal status, presence of metastases, nonsquamous histology, an elevated alkaline phosphatase level, a high serum CYFRA 21-1 level and a high serum NSE level). Neither HER-2 nor EGFR ECD specific levels were associated with a particular prognosis in this disease. Uncertainty regarding the prognostic effect of serum HER ECDs might depend on the poor reliability of these molecules as tumour markers.

One could object that, as functional markers, HER-2 and EGFR might be related to a particular prognosis only for those patients receiving specific targeted therapy. However, in the setting of EGFR-targeted therapy, two different studies of EGFR tissue expression failed to demonstrate any relationship between immunohistochemical staining intensity and clinical efficacy. Conversely, activity of anti-HER-2 therapy in lung cancer could be positively influenced by a 2+ or 3+ HER staining. The low frequency of such high expression in NSCLC weakens the clinical usefulness of this observation. Whether or not serum HER ECD levels could help in the management of specific targeted therapy remains to be evaluated. However, the recent discovery of a EGFR gene mutation as a predictive factor of response to gefitinib therapy seems a more reasonable and promising approach (Lynch *et al*, 2004).
